# Chemoimmunotherapy in the First-Line Treatment of Chronic Lymphocytic Leukaemia: Dead Yet, or Alive and Kicking?

**DOI:** 10.3390/cancers13133134

**Published:** 2021-06-23

**Authors:** Lukáš Smolej, Pavel Vodárek, Dominika Écsiová, Martin Šimkovič

**Affiliations:** 4th Department of Internal Medicine–Hematology, Faculty of Medicine, University Hospital, Charles University, 50005 Hradec Králové, Czech Republic; pavel.vodarek@fnhk.cz (P.V.); dominika.ecsiova@fnhk.cz (D.É.); martin.simkovic@fnhk.cz (M.Š.)

**Keywords:** chronic lymphocytic leukaemia, chemoimmunotherapy, direct inhibitors, ibrutinib, venetoclax, acalabrutinib, rituximab, obinutuzumab, prognosis

## Abstract

**Simple Summary:**

Chemoimmunotherapy has been the cornerstone of the first-line treatment for chronic lymphocytic leukaemia for almost a decade: FCR (fludarabine, cyclophosphamide, rituximab) or BR (bendamustine, rixutimab) regimens for fit patients and G-CLB (obinutuzumab, chlorambucil) being the most prominent examples. However, on the basis of several recent randomised phase III trials, chemoimmunotherapy is being replaced by treatment with regimens based on oral targeted inhibitors such as Bruton tyrosine kinase inhibitors ibrutinib and acalabrutinib, or bcl-2 inhibitor venetoclax. While these agents demonstrated significantly better efficacy than chemoimmunotherapy in terms of longer progression–free survival, the problems associated with their use include a specific spectrum of side effects, the need for long-term therapy, and a significant economic burden. This review focuses on the current role of chemoimmunotherapy in treatment-naïve patients with CLL.

**Abstract:**

The paradigm of first-line treatment of chronic lymphocytic leukaemia (CLL) is currently undergoing a radical change. On the basis of several randomised phase III trials showing prolongation of progression-free survival, chemoimmunotherapy is being replaced by treatment based on novel, orally available targeted inhibitors such as Bruton tyrosine kinase inhibitors ibrutinib and acalabrutinib or bcl-2 inhibitor venetoclax. However, the use of these agents may be associated with other disadvantages. First, with the exception of one trial in younger/fit patients, no studies have so far demonstrated benefit regarding the ultimate endpoint of overall survival. Second, oral inhibitors are extremely expensive and thus currently unavailable due to the absence of reimbursement in some countries. Third, treatment with ibrutinib and acalabrutinib necessitates long-term administration until progression; this may be associated with accumulation of late side effects, problems with patient compliance, and selection of resistant clones. Therefore, the identification of a subset of patients who could benefit from chemoimmunotherapy would be ideal. Current data suggest that patients with the mutated variable region of the immunoglobulin heavy chain (*IGHV*) achieve fairly durable remissions, especially when treated with fludarabine, cyclophosphamide, and rituximab (FCR) regimen. This review discusses current options for treatment-naïve patients with CLL.

## 1. Introduction

Chronic lymphocytic leukaemia (CLL), the most common lymphoid disorder in the European American population [1,2], is predominantly a disease of the elderly with the median age at diagnosis between 65 and 72 years [3,4,5,6,7,8,9,10]. CLL is characterised by an extreme heterogeneity of clinical course [11,12]. The majority of CLL patients have an indolent, asymptomatic course of the disease, while approximately 30–40% sooner or later require therapeutic intervention due to bone marrow failure, massive/progressive organomegaly, or systemic symptoms [3,4,7,13,14]. As the vast majority of CLL patients are nowadays diagnosed with early asymptomatic disease corresponding to Binet A/Rai 0–I clinical stages [15,16], it is valuable to refine an individual patient’s prognosis concerning the future risk of progression and treatment. There are numerous prognostic factors correlating with progressive clinical course in CLL but the mutational status of the immunoglobulin heavy chain variable region (*IGHV*) and cytogenetic aberrations detected by fluorescent in situ hybridisation (FISH) clearly belong to the most powerful ones [17,18,19,20]: patients with unmutated *IGHV* and/or unfavourable cytogenetic findings (deletion 11q or 17p) are much more likely to have progressive disease course. There are multiple prognostic scores which can be used to refine an individual patient´s prognosis at the time of CLL diagnosis [21]. Robust and externally validated systems include the CLL IPI project, the Barcelona–Brno score, and the MD Anderson nomogram [22,23,24]. Additionally, for patients diagnosed in Binet A stage, recently, two externally validated prognostic models have been developed based on the combination of IGVH/FISH with more traditional parameters such as absolute lymphocyte count and/or palpable lymphadenopathy [15,16,25,26]. More recently, complex karyotype (presence of ≥3 cytogenetic changes) has emerged as a potentially strong prognostic/predictive factor for unfavourable outcome, including the development of Richter´s transformation [27,28,29,30]; however, more validation using data from prospective clinical trials is needed to verify its classification (e.g., three vs. five changes as the cut-off) and prognostic/predictive value [31,32]. With regard to therapy, CLL had been for decades an uninteresting disease with little to do for the patients since various chemotherapeutic approaches failed to alter the natural course of the disease [33,34]; therefore, chlorambucil, an oral cytotoxic agent introduced into CLL therapy in the 1950s [35,36], remained the cornerstone of CLL therapy until the advent of regimens combining purine analogues, most notably fludarabine, with cyclophosphamide [37,38,39,40].

## 2. Chemoimmunotherapy

The era of chemoimmunotherapy (CIT) for the first-line therapy of CLL has been heralded by a phase II study investigating the combination of fludarabine, cyclophosphamide, and monoclonal anti-CD20 antibody rituximab (FCR) regimen developed at MD Anderson Cancer Centre in Houston, USA [41]. However, it was the seminal randomised phase III study CLL8 by the German CLL Study Group which conclusively demonstrated the superiority of FCR regimen over chemotherapy FC alone in terms of better complete response rate, eradication of minimal residual disease, and prolongation of progression-free survival (PFS), and for the first time in the modern history of CLL therapy, also prolongation of overall survival (OS) [42,43]. Updated long-term results of the CLL8 trial confirmed the findings of the initial publication and pointed out that FCR-treated patients with mutated *IGHV* (comprising 37% of the FCR arm) had extremely favourable prognosis considering PFS as well as OS, with approximately 55% without progression and approximately 82% alive at 7 years [44]; this has been corroborated by the MD Anderson single-centre analysis [45]. Nevertheless, FCR proved to be relatively toxic (inducing severe neutropenia in 34% and serious infections in 25%) even in the younger and fit CLL patients who comprise only about a third of the general CLL population requiring first-line treatment. Therefore, the subsequent CLL10 non-inferiority trial of the German CLL Study Group randomised FCR against the combination of bendamustine and rituximab (BR) which previously showed promising efficacy and safety in a phase II study [46]. FCR demonstrated better PFS than BR but at the expense of significantly higher toxicity (severe neutropenia 85 vs. 59%, serious infections in 40 vs. 26%). The difference in PFS was less pronounced in patients >65 years, leading to the recommendation of the BR regimen as an alternative to FCR in older fit patients [47]. Updated results of the CLL10 trial confirmed the sustained PFS benefit of FCR but no difference in OS (81 vs. 80% at 5 years); notably, secondary neoplasms occurred significantly more frequently with FCR in older patients (33 vs. 17%) [48].

Elderly/significantly comorbid patients, as noted above, represent the majority of the CLL population indicated for first-line therapy. However, the first phase III randomised study in this important clinical scenario emerged only in 2014 and, unsurprisingly, again from the German CLL Study Group: the CLL14 trial compared chlorambucil (CLB) monotherapy with rituximab + chlorambucil (R-CLB) and obinutuzumab (a second-generation glycosylated anti-CD20 antibody with enhanced antibody-dependent cellular cytotoxicity) + chlorambucil (G-CLB). The G-CLB turned out to be the winner, achieving significantly more complete responses, minimal residual disease (MRD) negativity, and prolonging PFS in comparison to both CLB monotherapy and R-CLB combination; significant toxicity associated with obinutuzumab was predominantly infusion-related reactions and neutropenia, without the increased occurrence of serious infections [49]. The updated publication reported even OS prolongation with G-CLB vs. R-CLB [50]. These results led to the G-CLB regimen being standard of care for elderly/comorbid CLL patients [51]. The addition of another second-generation antiCD20 antibody ofatumumab (with amplified complement-dependent cytotoxicity) to CLB was examined in the COMPLEMENT-1 phase III trial. The combination regimen achieved more CRs, MRD negativity, and longer PFS (median 22 vs. 13 months) [52]. However, the ofatumumab chlorambucil regimen did not gain widespread use, and ofatumumab was ultimately discontinued for commercial use in 2018. Another approach in the treatment of naïve elderly/comorbid patients was the utilisation of the BR regimen tested against R-CLB within the MABLE phase IIIb study. BR was associated with higher CR and MRD rates, as well as prolonged PFS; there was no difference in OS. While the incidence of severe neutropenia was similar (43 vs. 37%), more infections (19 vs. 8%) were recorded in the BR arm [53]. Finally, FCR with attenuated doses of chemotherapy was reported in smaller studies [54,55].

## 3. Targeted Oral Agents

The introduction of targeted oral agents interfering with key signalling pathways of the CLL cells revolutionised the treatment landscape of CLL.

### 3.1. Ibrutinib

Bruton tyrosine kinase (BTK) ibrutinib was the first in-class agent approved in the United States and European Union for the treatment of relapsed/refractory CLL following the successful results of the RESONATE randomised phase III trial showing significantly longer PFS and OS of ibrutinib vs. ofatumumab [56]. Ibrutinib subsequently achieved favourable results in terms of PFS and OS (despite the fact that crossover from control to experimental arm was not allowed) against CLB in treatment-naïve elderly/comorbid patients [57], but the results were published at the time when CLB monotherapy was no longer considered the standard of care due to the results of the CLL11 trial. The clinical development of ibrutinib in the first line proceeded with phase III randomised trials ILLUMINATE (G-CLB vs. G-ibrutinib) [58] and ALLIANCE (BR vs. ibrutinib vs. R-ibrutinib) for elderly/comorbid patients [59]; ECOG E-1912 (FCR vs. R-ibrutinib) for the younger, fit population [60]. All these studies (Table 1) showed significantly longer PFS in comparison to CIT; OS benefit was considered only in the E-1912 trial. Importantly, ibrutinib achieved excellent results in patients with mutation/deletion of the *TP53* gene [61,62,63], a molecular aberration well known for its association with resistance to CIT and extremely unfavourable clinical outcome [64,65,66,67]. While ibrutinib is generally well tolerated and induces less haematological toxicity than CIT, it is associated with a specific spectrum of side effects due to its off-target activity against other kinases. These side effects include cardiovascular problems (especially atrial fibrillation and arterial hypertension) and elevated risk of bleeding [68,69,70,71]. While most side effects are mild and manageable, 21% of patients who discontinued ibrutinib in the RESONATE-2 study did so because of toxicity, not progressive disease [72]; this number may be as high as 50% in the relapsed/refractory setting [68]. The necessity of long-term administration until progressive disease may also negatively impact patient compliance and lead to the selection of resistant clones; indeed, progression on ibrutinib is frequently associated with a mutation in the BTK or phospholipase Cg2 [73].

### 3.2. Idelalisib

The phosphatidylinositol-3-kinase delta (PI3K-δ) inhibitor idelalisib received approval on the basis of the 116 phase III randomised trial which reported longer PFS and OS of idelalisib + rituximab vs. placebo + rituximab [74]. Unfortunately, several idelalisib trials in the first-line treatment were terminated prematurely due to safety signals, specifically high occurrence of autoimmune complications (hepatotoxicity, colitis, pneumonitis) and infections, including the increase of treatment-associated mortality [75,76,77]. Therefore, idelalisib, though still having a label in the European Union for the first-line therapy (specifically, for patients with TP53 aberrations unsuitable for any other treatment), is virtually never used in this scenario; in addition, its utilisation in relapsed/refractory CLL is also declining due to higher toxicity and inferior efficacy in comparison to other targeted agents, as demonstrated by the ASCEND phase III trial showing longer PFS and better safety profile of acalabrutinib vs. idelalisib + rituximab [78].

### 3.3. Acalabrutinib

Acalabrutinib is a second-generation, highly selective BTK inhibitor designed to have fewer off-target effects, resulting in a better safety profile than ibrutinib [79,80]. Acalabrutinib received a registration for the treatment of CLL [81] due to positive results of two phase III randomised trials: ASCEND (acalabrutinib vs. investigator´s choice of idelalisib + rituximab or BR) in relapsed/refractory CLL [78], and ELEVATE-TN (acalabrutinib vs. obinutuzumab + acalabrutinib vs. G-CLB) in untreated elderly/comorbid patients [82]. In both of these studies, acalabrutinib proved more effective than the control arm in terms of longer PFS; notably, the side effect profile was very good with a lower occurrence of atrial fibrillation than in ibrutinib studies. The first head-to-head comparison of two BTK inhibitors was investigated in the ELEVATE-RR trial for relapsed/refractory CLL in which acalabrutinib demonstrated non-inferior PFS in comparison to ibrutinib (median 38 months in both arms), but there was a significantly lower incidence of atrial fibrillation with acalabrutinib (9 vs. 16%); among most frequent side effects, acalabrutinib also caused less hypertension, arthralgia, and diarrhoea but more headaches and cough. There were more discontinuations due to AEs with ibrutinib (21%) than acalabrutinib (15%) [83]. Finally, the long-term results of the phase I/II study in the untreated CLL population confirmed a very good safety profile, with less than 10% of patients discontinuing treatment due to an adverse event [84].

### 3.4. Venetoclax

Venetoclax represents a novel class of orally available selective inhibitors of bcl-2, specifically a BH3 mimetic which antagonises bcl-2 overexpression in CLL and thereby shifts the intracellular balance of bcl-2 activators and inhibitors towards the activation of apoptosis via the mitochondrial pathway. The molecule of venetoclax was developed following experience with its predecessor navitoclax which showed promising activity in CLL, but its further development in lymphoid malignancies was terminated because of dose-limiting thrombocytopenia as the result of BCL-XL inhibition in platelets [85,86]. Venetoclax showed promising activity in a dose-escalation phase I trial [87] and subsequently acquired registration for the treatment of relapsed/refractory CLL owing to results of a phase II trial in patients with 17p deletion [88]. Tumour lysis syndrome (TLS) emerged as the main serious early toxicity in a phase Ib trial combining venetoclax with rituximab; therefore, a careful ramp-up period with the starting dose of 20 mg was developed, which, together with strict prophylactic, measures corresponding to individual patient´s TLS risk, resulting in significantly reduced incidence of this side effect [89]. The first phase III randomised trial with venetoclax was the MURANO study comparing venetoclax–rituximab (VR) combination against BR in relapsed/refractory setting. Importantly, venetoclax was administered in a time-limited fashion for the maximum duration of 24 months. VR regimen achieved a higher rate of MRD negativity and significantly prolonged PFS and OS [90,91]; notably, the occurrence of TLS was low, at 3% (clinical TLS in one patient only). In the first-line scenario, venetoclax combined with obinutuzumab (VG) demonstrated superiority with regard to MRD negativity and PFS in comparison to the G-CLB regimen in elderly/comorbid patients (CLL14 phase III trial) [92,93].

## 4. Chemoimmunotherapy vs. Targeted Inhibitors

### 4.1. Efficacy

Very importantly, targeted oral inhibitors showed excellent efficacy in treatment-naïve patients with *TP53* mutation and/or deletion. This molecular abnormality is present in less than 15% of the patient population indicated for first-line therapy, but the outcome of classical chemoimmunotherapy regimens such as FCR, BR, or G-CLB in these patients has been dismal, with very low CR rate and short PFS and OS [47,49]. Therefore, chemoimmunotherapy is currently not recommended in patients with TP53 aberrations; ibrutinib or acalabrutinib, obinutuzumab + venetoclax, or idelalisib + rituximab should be used instead [94,95].

In younger/fit patients, there is only one study comparing CIT with novel inhibitors: the ECOG E-1912 which randomised in the 1:2 ratio between FCR and rituximab + ibrutinib (IR). The IR regimen achieved significantly longer PFS (at 3 years, 89 vs. 73%) and OS (at 3 years, 99 vs. 92%) in the whole patient population, while PFS was not significantly different between arms in patients with mutated *IGHV*. On the other hand, complete responses were more frequent in the FCR arm (30 vs. 17%), as was the MRD negativity rate (59 vs. 8%) [60]. Due to the results of two randomised studies showing little to no benefit of the addition of rituximab to ibrutinib [59,96], ibrutinib monotherapy, rather than IR, is recommended in the European Society for Medical Oncology (ESMO) and National Cancer Comprehensive Network (NCCN) guidelines [94,95]. Results of first-line regimens in younger/fit patients are summarised in Table 2.

In the elderly/comorbid population, two trials investigated ibrutinib regimens against CIT: the ILLUMINATE trial compared G-ibrutinib against G-CLB in a mix of older (>65) and comorbid patients; unfortunately, there was no ibrutinib monotherapy arm. While G-ibrutinib showed significantly longer PFS (at 30 months, 79 vs. 31%), more frequent CRs (19 vs. 8%), and MRD negativity (30 vs. 20% in peripheral blood), the uncertainty regarding the contribution of obinutuzumab to overall therapeutic effect and the fact that the patient cohort in this trial was less comorbid (median Cumulative Illness Rating Scale score 4) probably resulted in absence of the G-ibrutinib regimen among preferred first-line approaches, both in ESMO and NCCN guidelines [94,95]. The ALLIANCE trial evaluated ibrutinib vs. IR vs. BR, and two important observations were made: first, there was no benefit in adding rituximab to ibrutinib; second, ibrutinib was more effective than BR in terms of PFS (87 vs. 74% at 2 years), while OS was not significantly different; BR was associated with higher CR rate (26 vs. 7%) and MRD negativity (8 vs. 1%). The second-generation BTK inhibitor acalabrutinib was tested in monotherapy or its combination with obinutuzumab against G-CLB within the ELEVATE-TN trial. Rather surprisingly, the trial was not powered to detect the PFS difference between the acalabrutinib arms. Similar to the abovementioned studies, PFS was significantly longer with acalabrutinib vs. G-CLB (87 vs. 47% at 2 years); OS was comparable in all three arms. Complete responses were rare (G-CLB, 5%; acalabrutinib, 1%) [82]. The only randomised trial involving venetoclax in treatment-naïve patients was the CLL14 trial, randomising between venetoclax + obinutuzumab (VG) vs. G-CLB. Importantly (and unlike the other abovementioned trials), the duration of therapy was identical in both arms: venetoclax and chlorambucil were given for 12 cycles. Despite time-limited therapy, PFS was significantly longer with the VG regimen (at 3 years, 82 vs. 50%); CR rate (50 vs. 23%) and MRD negativity were also significantly better with VG (76 vs. 35%). There was no OS benefit associated with VG [92,93]. Results of first-line regimens in older patients are summarised in Table 3.

### 4.2. Toxicity

The safety profile of chemoimmunotherapy in CLL is rather well recognised. Neutropenia and infections belong to the most important side effects, with the highest rates logically associated with the most intensive FCR regimen (Table 2) [47]. Obinutuzumab is associated with a higher rate of infusion-related reactions and neutropenia than rituximab [49]. Bendamustine can be specifically associated with skin reactions (e.g., 13% of pts in the BR arm of the CLL 10 trial) [47] which are usually mild but rarely can present as Steven-Johnson syndrome requiring permanent discontinuation of bendamustine [97,98].

BTK inhibitors showed milder haematological toxicity in comparison to CIT; for example, severe neutropenia occurred in 26 vs. 45% with ibrutinib + rituximab vs. FCR [60], in 15 vs. 45% with ibrutinib vs. BR [59], or 10 vs. 41% with acalabrutinib vs. G-CLB [82]. Venetoclax typically causes a higher rate of severe neutropenia, e.g., 53% with VG vs. 48% with G-CLB [92]. The serious infection rate with the novel inhibitors was usually comparable to CIT, between 11 and 20% (Table 2, Table 3). Regarding other non-haematological toxicity, ibrutinib is associated with a specific spectrum of side effects which is quite different from that of CIT regimens. While the most frequent, usually mild side effects include diarrhoea, rash, and skin bleeding, the major safety issues with ibrutinib have been cardiovascular side effects, namely, atrial fibrillation (AF), occurring in the first line in 4–17% of patients (a prognostic score aiming at prediction of AF in ibrutinib-treated patients has been developed [99]), and severe haemorrhage in 1–2% [58,59,60]. Acalabrutinib appears to have a more favourable safety profile, especially a lower incidence of atrial fibrillation (4% in the ELEVATE-TN trial) [82]. Finally, there is a substantial risk of drug interactions with novel inhibitors due to the fact that these agents are metabolised in the liver [71,100,101].

### 4.3. Financial Burden, Availability

**Figure 1:**
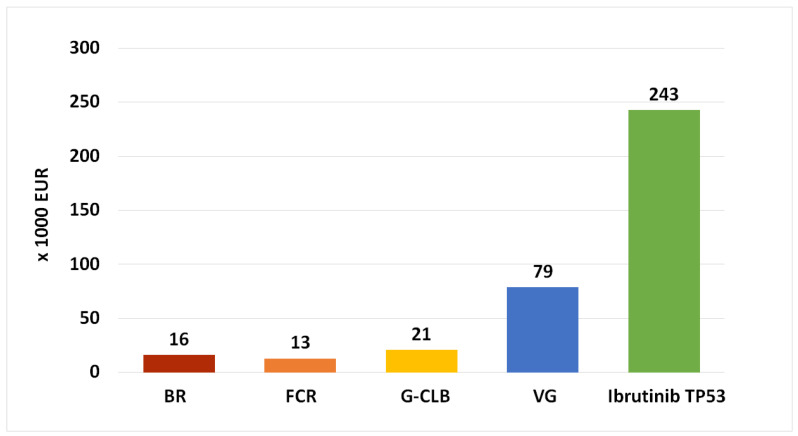
Gross cost of first-line CLL treatment (drugs only), Czech Republic, 2021. The price is in thousands of EUR and reflects median duration of therapy in the published studies. Venetoclax is currently not reimbursed for the first-line CLL therapy in the Czech Republic. G-CLB, obinutuzumab + chlorambucil; BR, bendamustine + rituximab; FCR, fludarabine + cyclophosphamide + rituximab; VG, venetoclax + obinutuzumab.

Oral targeted inhibitors are more expensive than classical CIT in terms of healthcare budget impact/economic burden [102,103,104]. Some of the reasons for this fact include the cost of drug development, lack of transparency, and lack of free-market competition [105]. Figure 1 shows the gross cost of different CIT vs. targeted inhibitor regimens in the Czech Republic and illustrates the unprecedented rise of the expenses associated with novel oral inhibitors. The main disadvantage of BTK inhibitors ibrutinib and acalabrutinib in this regard is the need for long-term administration until progression or unacceptable toxicity. For example, the median duration of initial ibrutinib therapy in 89 patients with *TP53* aberration (an indication which, due to the highest benefit vs. CIT, has the widest international availability in terms of reimbursement) was 46 months [63]; median treatment duration with acalabrutinib in the ELEVATE-TN was 28 months [82]. Several publications dealing with the economic burden and cost-effectiveness of ibrutinib concluded that despite undeniable excellent efficacy, ibrutinib was not cost-effective in comparison to chemoimmunotherapy [106,107]. Indeed, a recent study estimated that ibrutinib used in the first-line scenario was associated with the cost of USD 2.35 million per quality-adjusted life-year (QALY) and so would have to be cheaper by 72% in order to be cost-effective by reaching the willingness-to-pay (WTP) threshold of 150,000 USD/QALY, as accepted in the United States [108]; the WTP in European countries is considerably less, e.g., roughly USD 42,000 (GBP 30,000) in the United Kingdom. In contrast, the obinutuzumab–venetoclax combination, approved on the basis of the CLL14 study, utilises the time-limited approach, with venetoclax therapy limited to the maximum of 12 months [92]. A recent analysis concluded that the VG regimen is more cost-effective than ibrutinib or BR in the first-line scenario [109]. Due to financial demands and issues regarding their cost-effectiveness, the availability of novel inhibitors is limited in multiple EU countries according to a recent electronic survey (Tadeusz Robak, personal communication; Carol Moreno, personal communication; Stefano Molica, personal communication; Zoltán Mátrai, personal communication).

## 5. Conclusions

While regimens based on oral targeted inhibitors demonstrated better results in terms of longer PFS, there was no benefit (except for one study: ECOG E-1912 in younger/fit pts) regarding OS, the ultimate endpoint. Importantly, patients with mutated *IGHV* gene represent a subgroup, which appears to have the greatest benefit from this approach, especially with the FCR regimen, due to very good results regarding PFS [110]. Additionally, the huge increase in treatment cost incurred by oral targeted inhibitors represents a significant economic burden, thus negatively affecting the real-life availability of these agents, especially in countries with significant healthcare budget constraints. Therefore, it seems that chemoimmunotherapy is not dead yet but remains an important therapeutic approach for untreated CLL. Further research on the role of CIT vs. oral targeted agents in the subgroup of *IGHV*-mutated patients, ideally via biomarker-driven phase III randomised trials, as already employed in diffuse large B-cell lymphoma [111], would be highly beneficial.

## Figures and Tables

**Table 1:** Phase III randomised trials comparing chemoimmunotherapy with novel agents in the first-line of CLL. * mean; R, rituximab; G, obinutuzumab; CLB, chlorambucil; BR, bendamustin + rituximab; FCR, fludarabine + cyclophosphamide + rituximab; ibru, ibrutinib; acala, acalabrutinib; y, years; m, months; MRD, minimal residual disease; PFS, progression-free survival; OS, overall survival; NR, not reported.

Study	Patient Population	Study Design	Crossover	Median Age	MRD neg.	PFS	PFS Benefit	OS	OS Benefit	Reference
ECOG 1912	Younger/fit	R-ibru vs. FCR	No	57 vs. 57 *	8 vs. 59%	3y: 89 vs. 72%	Yes	3y: 99 vs. 92%	Yes	Shanafelt 2019
CLL14	Elderly/comorbid	G-venetoclax vs. G-CLB	No	72 vs. 71	76 vs. 35%	3y: 82 vs. 50%	Yes	3y: 88 vs. 87%	No	Fischer 2019; Al-Sawaf 2020
ILLUMINATE	Elderly/comorbid	G-ibru vs. G-CLB	Yes	70 vs. 72	30 vs. 20%	30m: 79 vs. 31%	Yes	30m: 86 vs. 85%	No	Moreno 2019
ELEVATE-TN	Elderly/comorbid	G-acala vs. acala vs. G-CLB	Yes	70 vs. 70 vs. 71	NR	2y: 93 vs. 87 vs. 47%	Yes	2y: 95 vs. 95 vs. 92%	No	Sharman 2020
ALLIANCE	Elderly/comorbid	R-ibru vs. ibru vs. BR	Yes	71 vs. 71 vs. 70	4 vs. 1 vs. 8%	2y: 88 vs. 87 vs. 74%	Yes	2y: 94 vs. 90 vs. 95%	No	Woyach 2018

**Table 2:** Results of first-line randomised trials in younger/fit patients. * FCR and FCM-miniR analysed together. BR, bendamustine + rituximab; FCR, fludarabine + cyclophosphamide + rituximab; CrCl, creatinine clearance; ORR/CR, response rate/complete responses; CIRS, Cumulative Illness Rating Scale; PFS, progression-free survival; NR, not reported; *IGHV*, variable region of immunoglobulin heavy chain; FISH, fluorescent in situ hybridisation.

Variable	BR CLL10	FCR CLL8	FCR CLL10	FCR ARCTIC	FCR ECOG/ACRIN	IR ECOG/ACRIN
*n*	279	408	282	100	175	354
Median age	61	61	62	63	57 (mean)	57 (mean)
Median CrCl (mL/min)	86	NR	87	NR	NR	NR
Median CIRS	2	1	2	NR	NR	NR
Unmutated *IGHV*, %	68	63	55	52	62	75
FISH del 11q, %	23	22	24	10	22	22
FISH del 17p, %	0	10	0	4	0	1
ORR/CR, %	96/31	90/44	95/40	94/68	81/30	96/17
Median PFS, months	42	52	58	58	not reached, 73% at 3y	not reached, 89% at 3y
Median PFS, M-*IGHV*	69	not reached, 67% at 5y	not reached, 65% at 5y	not reached, 68% at 5y *	not reached, 88% at 3y	not reached, 88% at 3y
Neutropenia grade 3–4	59	34	85	14	45	26
Infections grade 3–5	26	25	40	58	20	11
Reference	Eichhorst, 2016; Kutsch, 2020	Hallek, 2010; Fischer, 2016	Eichhorst, 2016; Kutsch, 2020	Howard, 2017	Shanafelt, 2019	Shanafelt, 2019

**Table 3:** Results of first-line randomised trials in elderly/comorbid patients. G-CLB, obinutuzumab + chlorambucil; BR, bendamustine + rituximab; VG, venetoclax + obinutuzumab; CrCl, creatinine clearance; *IGHV*, variable region of immunoglobulin heavy chain; M, mutated; NA, not available; NR, not reached; FISH, fluorescent in situ hybridisation; ORR/CR, overall response rate/complete responses; CIRS, Cumulative Illness Rating Scale; PFS, progression-free survival.

Variable	G-CLB CLL11	G-CLB CLL14	G-CLB ILLUMINATE	G-CLB ELEVATE-TN	BR MaBLe	BR ALLIANCE	Ibrutinib ALLIANCE	G-ibrutinib ILLUMINATE	Acalabrutinib ELEVATE-TN	VG CLL14
*n*	238	216	116	177	121	113	182	113	179	216
Median age	74	71	72	71	72	70	71	70	70	72
Median CrCl (mL/min)	61	66	70	70	NA	67	69	72	75	65
Median CIRS	8	8	4	6	3 comorbidities	2 comorbidities	2 comorbidities	4	6	9
Unmutated *IGHV*, %	61	59	53	66	60	58	63	62	58	59
FISH del 11q, %	16	18	19	19	20	18	19	12	17	17
FISH del 17p, %	8	7	16	9	8	8	5	12	10	8
ORR/CR, %	78/21	71/23	73/8	79/5	91/24	81/26	93/7	88/19	86/1	85/50
Median PFS, months	32	36	22	23	40	43	NR; 87% at 2y	NR; 80% at 2y	NR; 87% at 2y	NR; 82% at 3y
Median PFS M-*IGHV*, months	NA	43	NR; 63% at 2y	NR; 68% at 3 y	NA	51; 74% at 4y	NR; 83% at 4y	NR; 87% at 2y	NR; 80% at 3y	NR; 88% at 3y
Neutropenia grade 3–4	33	48	46	41	43	40	15	37	10	53
Infections grade 3–5	12	15	11	8	19	15	20	16	14	17
Reference	Goede 2014; Goede 2015	Fischer 2019; Al-Sawaf 2020	Moreno 2019	Sharman 2020	Michallet 2018	Woyach 2018	Woyach 2018	Moreno 2019	Sharman 2020	Fischer 2019; Al-Sawaf 2020
